# NIKEI: A New Inexpensive and Non-Invasive Scoring System to Exclude Advanced Fibrosis in Patients with NAFLD

**DOI:** 10.1371/journal.pone.0058360

**Published:** 2013-03-26

**Authors:** Münevver Demir, Sonja Lang, Martin Schlattjan, Uta Drebber, Inga Wedemeyer, Dirk Nierhoff, Ingrid Kaul, Jan Sowa, Ali Canbay, Ulrich Töx, Hans-Michael Steffen

**Affiliations:** 1 Clinic for Gastroenterology and Hepatology, University Hospital of Cologne, Cologne, Germany; 2 Clinic for Gastroenterology and Hepatology, University Hospital of Essen, Essen, Germany; 3 Institute for Pathology, University Hospital of Cologne, Cologne, Germany; 4 Institute of Medical Statistics, Informatics and Epidemiology, University Hospital of Cologne, Cologne, Germany; Institute of Hepatology, Foundation for Liver Research, United Kingdom

## Abstract

**Aims:**

To develop, validate and compare a non-invasive fibrosis scoring system for non-alcoholic fatty liver disease (NAFLD) derived from routinely obtained clinical and biochemical parameters.

**Methods:**

267 consecutive patients with biopsy proven fatty liver or non-alcoholic steatohepatitis were randomly assigned to the estimation (2/3) or validation (1/3) group to develop a model for the prediction of advanced fibrosis. Univariate statistics were performed to compare patients with and without advanced fibrosis, and following a multivariate logistic regression analysis a new scoring system was constructed. This non-invasive Koeln-Essen-index (NIKEI) was validated and compared to the FIB-4 index by calculating the area under the receiver operating characteristic curve (AUC). We evaluated a stepwise combination of both scoring systems for the precise prediction of advanced fibrosis. To set in contrast, we additionally tested the diagnostic accuracy of the AST/ALT ratio, BARD score and the NAFLD fibrosis score in our cohort.

**Results:**

Age, AST, AST/ALT ratio, and total bilirubin were identified as significant predictors of advanced fibrosis and used to construct the NIKEI with an AUC of 0.968 [0.937; 0.998] compared to 0.929 [0.869; 0.989] for the FIB-4 index. The absence of advanced fibrosis could be confirmed with excellent accuracy (99–100%). The positive predictive value of the FIB-4 index was higher (100% vs. 60%), however, the false negative rate was also high (33%). With a stepwise combination of both indices 82%–84% of biopsies would have been avoidable without a single misclassification. The AUROC for AST/ALT ratio, the NAFLD fibrosis score, and the BARD score were 0.81 (95% CI, 0.72–0.90), 0.96 (95% CI 0.92–0.99), and 0.67 (95% CI 0.55–0.78), respectively.

**Conclusion:**

The NIKEI can reliably exclude advanced fibrosis in subjects with NAFLD. In combination with the FIB-4 index misclassification with inadequate clinical management can be avoided while the need for liver biopsies can be reduced.

## Introduction

Non-alcoholic fatty liver disease (NAFLD) has become the leading cause of chronic liver disease with prevalence rates ranging from 6% to 35% worldwide [1]. It is typically associated with type 2 diabetes mellitus (T2DM), hypertension, obesity, or dyslipidemia and can be considered as the hepatic manifestation of the metabolic syndrome [2]. NAFLD encompasses a histological spectrum ranging from simple steatosis (NAFL) to non-alcoholic steatohepatitis (NASH) with subsequent cirrhosis. Recent findings even suggest progression of NASH to hepatocellular carcinoma, in the absence of cirrhosis. While simple steatosis carries a relatively benign prognosis, the risk of cardiovascular as well as liver-related events is increased in individuals with NASH [3], and in a recently published study advanced fibrosis demonstrated the best independent association with long-term liver-related mortality in patients with NAFLD [4].

Currently, percutaneous liver biopsy is considered as the gold standard for the assessment of hepatic fibrosis and inflammation in chronic liver disease. However, liver biopsy is an invasive procedure with associated costs, complications, and inherent inaccuracy due to sampling error and inter- and intraobserver variability in histopathological interpretation [5], [6]. Therefore, it has been attempted to establish non-invasive methods for the detection of liver fibrosis. Various biochemical markers reflecting inflammatory activity [7]–[11], activity of apoptosis [12], and oxidative stress [13] have been evaluated. None of these markers has proven sufficient diagnostic accuracy and numbers of cases were rather low [14]. Furthermore, several scoring systems and specialized biomarkers for the prediction of fibrosis in NAFLD have been developed by combining various serologic and clinical parameters [15]–[22]. However, even with the best non-invasive simple scoring systems, 11% to 26% of patients with advanced fibrosis will be misclassified as no or mild fibrosis leading to an inappropriate clinical management [23]. Additionally, some of the parameters used in these scoring systems are not available on a routine basis.

As long as there are no approved therapeutic modalities it seems reasonable to target the clinical management at those patients most in need for surveillance and monitoring of complications. Thus, the aim of this study was (*i*) to define independent predictors of advanced fibrosis in a large cohort of patients with biopsy-proven NAFLD from two separate tertiary care medical centers in Germany (University hospitals of Cologne and Essen), (*ii*) to develop and validate a simple noninvasive fibrosis scoring system for NAFLD derived from routinely obtained and easily available clinical and biochemical parameters, and (*iii*) to compare the diagnostic accuracy and clinical utility of this noninvasive Koeln-Essen-index (NIKEI) alone or in combination with the FIB-4 index, a validated scoring system for the prediction of advanced fibrosis in NAFLD [19]. To set in contrast to these results, we aimed to analyze the diagnostic performance and clinical utility of the AST/ALT ratio, BARD score [22] and the NAFLD fibrosis score [17] in our study population.

## Patients and Methods

### Study population

Data from 408 consecutive patients with suspected NAFL/NASH who presented to the Clinic for Gastroenterology and Hepatology, University Hospital of Cologne (n = 323) or to the Clinic for Gastroenterology and Hepatology, University Hospital of Essen (n = 85) between July 1998 and November 2009, were analyzed retrospectively. Written informed consent from the participants and a votum of the local ethics committee were not obtained. In accordance with German law, a votum of a local ethics committee is not required to conduct strictly retrospective studies (paragraph 15, sentence 1, Nordrhein Medical Association's professional code of conduct from 14 November 1998 as amended on 19 November 2011). There is also no need to get written informed consent from the participants if the considered data was collected and analysed retrospectively (paragraph 6, sentence 1, Health Data Protection Act of Nordrhein-Westfalen).

While almost all patients in Cologne had been referred to the outpatient department for further work-up after abnormal liver function tests had been detected by their treating primary care physicians, patients in Essen had been recruited at the Department II of Surgery, Alfred-Krupp Hospital Essen or the Clinic for Surgery, Visceral and Vascular Surgery, Protestant Hospital Dinslaken. Patient charts were reviewed for the following parameters: age, gender, body height, body weight, arterial blood pressure, pulse rate, concomitant diseases and medication, alcohol consumption, serology for chronic viral infections (HBV, HCV, HIV), and autoimmune liver disease (ANA, AMA, Anti-SMA, Anti-LKM1, Anti-SLA, p-ANCA). In addition, laboratory parameters (hemoglobin, WBC, platelet count, aspartate aminotransferase (AST), alanine aminotransferase (ALT), gamma glutamyl transferase (GGT), alkaline phosphatase, total bilirubin, cholinesterase, creatinine, urea, glomerular filtration rate according to the MDRD formula, albumin, quantitative immunoglobulins, ferritin, urinary copper excretion, coeruloplasmin, triglycerides, total blood cholesterol, high-density lipoprotein cholesterol, low-density lipoprotein cholesterol, prothrombin time, INR, prothrombin time, fasting glucose, and alpha-fetoprotein (AFP)), histological data of liver biopsies, and results of abdominal ultrasonography as well as number and date of follow-up visits were documented. All laboratory parameters were taken under fasting conditions.

Arterial hypertension was defined as a blood pressure ≥140/90 mmHg in the medical record or a history of taking antihypertensive drugs. The diagnosis of concomitant diseases such as diabetes mellitus, arterial hypertension or dyslipidemia was based on findings from patient charts.

A diagnosis of NAFLD was made if the following conditions were met: elevated aminotransferase levels for at least six months, fatty liver degeneration >5% and if applicable additional inflammation, after exclusion of other chronic liver diseases, e.g. viral hepatitis, autoimmune hepatitis, toxic liver injury, alcoholic steatohepatitis, cholestatic liver disease, hemochromatosis. Patients were excluded if they suffered from a malignancy, had decompensated liver cirrhosis or received drugs with well-known effects on steatosis, e.g. methotrexate or glucocorticoids. They were also excluded if the time interval between liver biopsy and date of laboratory examination exceeded 120 days or if data to definitely exclude other chronic liver diseases were missing. Patients were included if alcohol consumption was less than 30 g/d in men and 20 g/d in women. According to these criteria, 141 patients had to be excluded from our study leaving 267 patients for further analyses.

### Liver histology

All liver specimens were taken under local anesthesia (17G Hepafix® Menghini type needle, Fa. Braun, Melsungen, Germany) at the Clinic for Gastroenterology and Hepatology, University of Cologne, or during surgery at the Department II of Surgery, Alfred-Krupp Hospital Essen or the Clinic for Surgery, Visceral and Vascular Surgery, Protestant Hospital Dinslaken and were assessed at the Institute for Pathology, University of Cologne. Liver biopsies were read twice by two experienced liver pathologists (D.U., W.I.), who were blinded for all clinical and laboratory patient data. Discordant ratings between these two were solved by majority decision. The stage of fibrosis was scored based on the 5-point scale proposed by Brunt et al. [24] and modified by Kleiner et al. [25].

### Statistical analysis and model building

The aim of the study was the development of a prediction model for advanced fibrosis (F3–F4) by applying routinely measured clinical and laboratory parameters. Patients from both centers were pooled, and 2/3 were then randomly assigned to the estimation group (n = 170) for model building and the remaining to the validation group (n = 97). The Kolmogorov-Smirnov test was used to test for normal distribution and means ± SD were calculated for continuous variables, frequencies for categorical variables. Univariate statistics were performed to look for differences between the estimation and validation groups and to compare patients with (F3–F4) and without (F0–F2) advanced fibrosis using the Student's t-test or the *X* ^2^-test. All variables with significant differences between the two fibrosis groups (p-value <0.05) were included in a multivariate logistic regression analysis to identify variables predicting the presence or absence of advanced fibrosis. Variables with p-values <0.05 in the regression analysis were then used to construct a new scoring system to predict advanced fibrosis. The overall diagnostic accuracy of this noninvasive Koeln-Essen-index (NIKEI) was determined by calculating the area under the receiver operating characteristic curve (AUROC) with its 95% confidence intervals. Validation was performed in the subset of patients with complete data (n = 92) from the validation group. From the ROC curve of the final model, two cutoff points were selected, with sensitivity and specificity for advanced fibrosis of at least 90%. The diagnostic accuracy of the two cutoff points was determined by calculating sensitivity, specificity, PPV, NPV, and likelihood ratios. Finally, AUROC with 95% confidence intervals (95% CI) was employed to compare performance of the NIKEI, the FIB-4 index the NAFLD fibrosis score, the AST/ALT ratio and the BARD score using previously published cut-off levels [17]; [22]; [26]. All analyses were performed with SPSS Statistics 19.0.0 (Release 19.0 SPSS Inc. Chicago, USA).

## Results

### Characteristics of study cohort

The demographic and laboratory characteristics of all included patients (n = 267) are shown in table 1. There were no statistically significant differences between the estimation and validation groups. 218 patients (82%) were overweight or obese, and 245 patients (92%) had no or mild fibrosis stages (F0–F2). As the identification of patients with advanced fibrosis is of clinical importance, the clinical and laboratory features of subjects with no/mild fibrosis (F0–F2) were compared to those with advanced fibrosis (F3–F4). The univariate analysis in the estimation group showed that patients with advanced fibrosis were significantly older, had lower platelet counts and albumin levels (p<0.001 in all cases), while values for AST (p<0.001), the AST/ALT ratio (p = 0.001), GGT (p<0.001), total bilirubin (p = 0.016), INR (p = 0.041), glucose (p<0.001, and AFP (p = 0.020) were all significantly increased.

**Table 1:** Characteristics of the study population.

Variable	All patients	Estimation G.	Validation	Koeln	Essen
	(n = 267)	(n = 170)	(n = 97)	(n = 183)	(n = 84)
**Age (years)**	43.8±12.1	43.9±11.9	43.7±12.6	44.8±12.6	41.8±10.8
**Female n, (%)**	141 (52.8)	89 (52.4)	52 (53.6)	70 (38.3)	71 (84.5)
**BMI (kg/m^2^)**	37.0±12.7	37.2±13.0	36.6±12.9	29.0±6.1	52.4±7.6
**BMI normal/overweight/obese, n**	25/79/139	13/53/91	12/26/48	25/79/56	0/0/82
**Stage of fibrosis 0/1/2/3/4, n**	8/107/130/18/4	7/68/82/11/2	1/39/48/7/2	8/91/63/17/4	0/16/67/1/0
**Hypertension n, (%)**	112 (41.9)	74 (43.5)	38 (39.6)	73 (39.9)	39 (46.4)
**Diabetes n, (%)**	53 (19.9)	33 (19.4)	20 (20.6)	19 (10.4)	34 (40.5)
**Platelet (1.000/µl)**	265.0±80.0	265.4±80.9	264.3±78.7	247.8±71.5	299.0±85.2
**AST (IU/ml)**	36.8±32.4	34.9±22.8	40.2±44.7	40.9±37.5	27.9±13.5
**ALT (IU/ml)**	56.6±55.2	54.8±44.4	59.8±70.5	66.4±62.6	35.5±22.8
**AST/ALT ratio**	0.8±0.3	0.8±0.4	0.7±0.3	0.7±0.3	0.9±0.3
**<1,0 n, (%)**	214 (80.5)	135 (79.4)	79 (82.3)	154 (84.2)	60 (71.4)
**≥1,0 n, (%)**	52 (19.5)	35 (20.6)	17 (17.7)	28 (15.4)	24 (28.6)
**GGT (IU/ml)**	111.4±186.1	128.8±222.7	80.5±82.8	136.8±201.2	56.2±133.4
**Total bilirubin (mg/dl)**	0.7±0.7	0.7±0.9	0.6±0.4	0.8±0.9	0.5±0.3
**Prothrombin time %**	101.5±14.6	100.3±15.8	103.8±11.9	103.7±13.5	97.0±15.8
**INR**	1.0±0.10	1.0±0.1	1.0±0.1	1.0±0.1	0.97±0.1
**Creatinine (mg/dl)**	0.9±0.7	0.9±0.8	0.8±0.2	0.9±0.8	0.8±0.5
**Albumin (g/l)**	44.6±3.4	44.8±3.5	44.4±3.3	44.6±3.4	no data
**Glucose (mg/dl)**	102.8±30.6	103.1±32.6	102.3±26.8	102.8±30.6	110.9±48.7
**Alkaline phosphatase (IU/ml)**	111.9±52.9	114.1±57.5	108.1±43.8	111.9±52.9	no data
**AFP (kU/l)**	3.0±1.8	3.1±2.0	2.7±1.4	3.0±1.8	no data

Data are shown as mean ± SD. Percentages (%) are given in brackets and refer to patients inside of respective subset. Abbreviations: BMI  =  body mass index; AST  =  aspartate aminotransferase; ALT  =  alanine aminotransferase; GGT  =  gamma glutamyl transferase; INR  =  International normalized ratio; AFP  =  alpha-fetoprotein.

### Generation of the NIKEI

**Figure 1:**
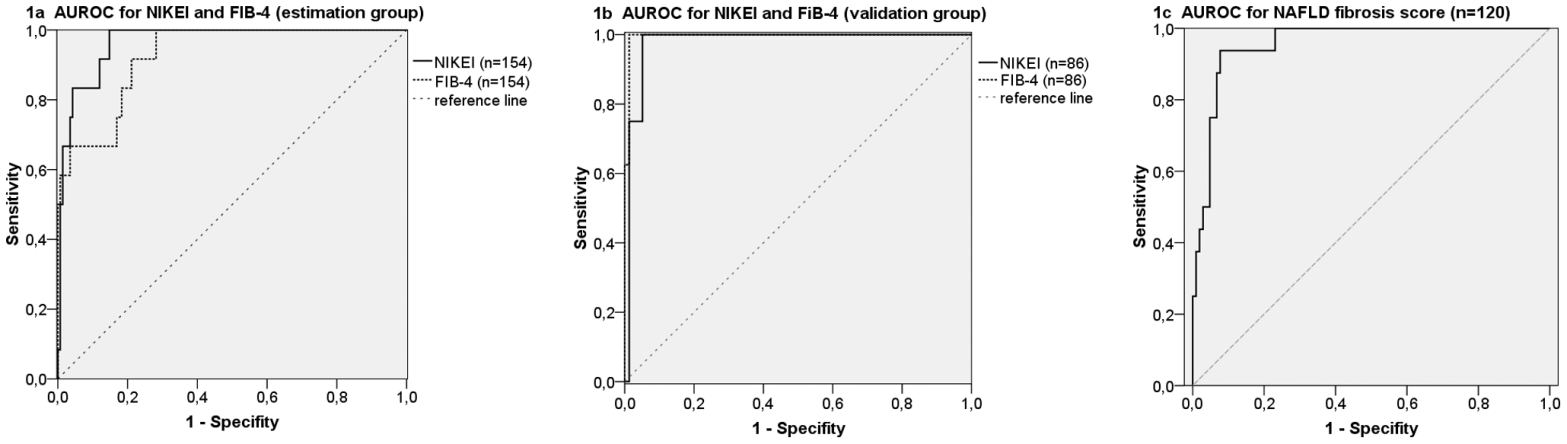
ROC-curve analysis for the prediction of advanced fibrosis with NIKEI, FIB-4 index and NAFLD fibrosis score. a) estimation group (n = 154 with complete data for calculating both indices); b) validation group (n = 86 with complete data for calculating both indices). c) NAFLD fibrosis score (n = 120).

By multivariate analysis, four variables remained significant including age, AST, AST/ALT ratio, and total bilirubin. No statistically significant interactions were identified. Using these four variables, we constructed an index to calculate the probability P of advanced fibrosis:
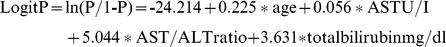



The area under the ROC curve (AUROC) for this NIKEI was 0.968±0.016 (95% CI: 0.937, 0.998) that compared favorable to the well-established FIB-4 index: 0.929 (0.869, 0.989; see figure 1a). From this ROC curve two cutoff points were selected for the regression function to identify the presence (≥0.2294) or absence (≤0.0535) of advanced fibrosis.

### Comparison of NIKEI and FIB-4 demonstrates superior NPV for NIKEI

By applying the lower cutoff point (≤0.0535), 128 (85%) of the 150 patients without advanced fibrosis were correctly identified. However, the absence of advanced fibrosis could be confirmed with excellent accuracy (NPV of 100%). In contrast, although the positive predictive value of the FIB-4 index for the prediction of advanced fibrosis was higher (100%), the false negative rate was 4 out of 12 patients.

### Evaluation of the NIKEI in separate patient cohort supports high NPV

The diagnostic accuracy of the NIKEI was then cross-validated in a separate set of 92 patients from the validation group. Results were similar to those of the estimation group. The AUROC was 0.961±0.023 (95% CI: 0.916, 1.000), again comparing favorably to the well-established FIB-4 index (see figure 1b). By applying the lower cutoff point (≤0.0535), 74 (89%) of the 83 patient without advanced fibrosis were correctly staged, whereas 3 (3%) were misclassified as having advanced fibrosis. 6 of 9 patients (67%) with values above the higher cutoff point (≥0.2294) were correctly identified as having advanced fibrosis. However, one patient (11%) with advanced fibrosis was misclassified (table 2). As in the estimation group, the positive predictive value of the FIB-4 index for the prediction of advanced fibrosis was higher (100%).

**Table 2:** NIKEI test performance in estimation group (EG) and validation group (VG).

	≤0.0535		0.0536–0.2293		≥0.2294		All patients	
	EG	VG	EG	VG	EG	VG	EG	VG
**All patients n**	128	75	19	8	15	9	162	92
**F0–F2 n**	128	74	16	6	6	3	150	83
**F3–F4 n**	0	1	3	2	9	6	12	9
**Sensitivity %**	100	88.9			75.0	66.7		
**Specifity %**	85.3	89.2			96.0	96.4		
**PPV %**	35.3	47.1			60.0	66.7		
**NPV %**	100	98.7			98.0	96.4		
**Likelihood Ratio (+)**	6.8	8.2			18.8	18.5		
**Likelihood-Ratio (−)**	0.0	0.1			0.3	0.3		
**AUC (95% CI)** EG: 0.968±0.016 (0.937; 0.998) VG: 0.961±0.023 (0.916; 1.000)								

Cut-off levels and the classification of patients on the basis of the individual scores within the estimation (EG) and validation group (VG). A patient with a value below the lower cut-off level was classified as healthy (no advanced fibrosis), with a value above the upper cut-off level as morbid (advanced fibrosis). Patients with values between the cut-off levels are intermediate/not classifiable.

### Stepwise scoring with FIB-4 and NIKEI enhances overall performance

**Figure 2:**
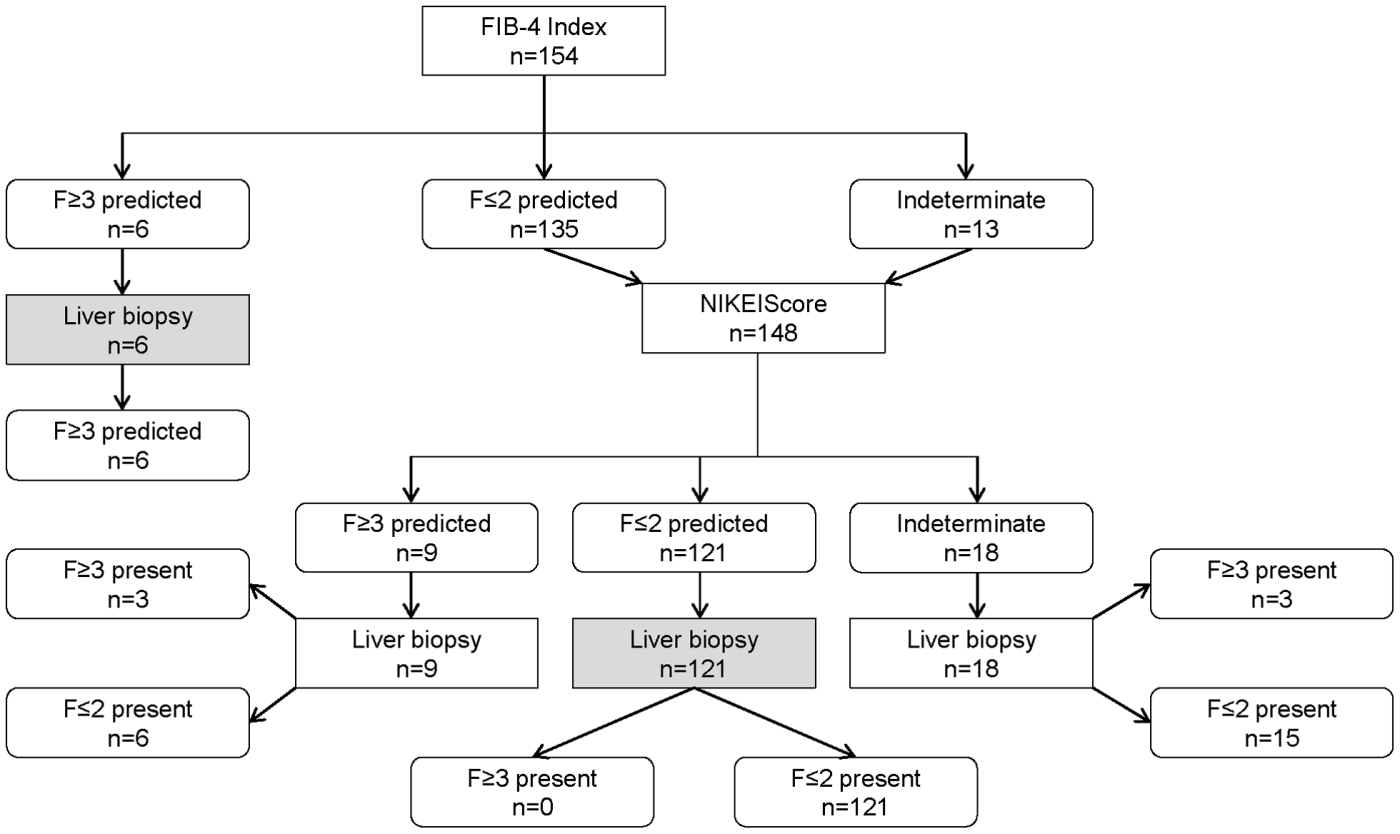
Stepwise combination of FIB-4 index and NIKEI. Algorithm for the prediction of advanced fibrosis in NAFLD patients (estimation group, n = 154 with complete data for calculating both indices). Algorithm starts with the calculation of the FIB4 index followed by calculating NIKEI only for patients with negative or indeterminate test results. Shaded rectangles: liver biopsies which could have been avoided.

We then evaluated a stepwise combination of both scoring systems in a subgroup of 154 patients with complete data for calculating both indices, i.e. the FIB-4 index first, followed by the NIKEI only for patients with indeterminate or negative prediction of advanced fibrosis. Assuming that a liver biopsy reasonably could have been avoided in patients with test results above (presence of advanced fibrosis) or below (absence of advanced fibrosis) the respective cut-offs, 82% of biopsies could have been omitted without missing a single case of advanced fibrosis (figure 2). Also, combining both scoring systems in the same stepwise fashion in the validation group would have allowed avoiding liver biopsy in 84% of cases without a single misclassification (not shown).

### Evaluation of the NAFLD fibrosis score in our study population

Additionally we calculated the NAFLD fibrosis score in our cohort and then compared to the NIKEI index (see table 3). Although AUROC and NPV showed similar results compared to the NIKEI index, the false negative rate was 25% for the NAFLD fibrosis score. However, only 120 patients were included to test the NAFLD fibrosis score because of incomplete data.

**Table 3:** Diagnostic performance of the NAFLD score.

NAFLD score	≤−1.455	−1.454 −0.675	≥0.676	All patients
**All patients, ** ***n (%)***	101 (84)	16 (13)	3 (3)	120
**F0–F2, ** ***n (%)***	97 (93)	7 (7)	0	104
**F3–F4, ** ***n (%)***	4 (25)	9 (56)	3 (19)	16
**Sensitivity, ** ***%***	75		19	
**Specifity, ** ***%***	93		100	
**PPV, ** ***%***	63		100	
**NPV, ** ***%***	96		89	
**Likelihood-Ratio (+)**	11.2		∞	
**Likelihood-Ratio (−)**	0.27		0.81	
**Diagnostic accuracy** 0.83				
**AUC (95% CI)** 0.96 (0.92; 0.99)				

Cut-off values and the classification of patients on the basis of the NAFLD fibrosis score (n = 120). A patient with a value below the lower cut-off level was classified as healthy (no advanced fibrosis), with a value above the upper cut-off level as morbid (advanced fibrosis). Patients with values between the cut-off levels are intermediate/not classifiable.

### Evaluation of the AST/ALT ratio and the BARD score in our study population

We also evaluated the AST/ALT ratio and the BARD score in our cohort. The AUROC were as follows: AST/ALT ratio 0.81 (95% CI, 0.72–0.90), and BARD score 0.67 (95% CI 0.55–0.78) with negative predictive values for advanced fibrosis of 96% and 95%, respectively (see table S1 and figure S1).

## Discussion

Liver fibrosis results from chronic injury [3], and in the absence of approved treatment modalities for NAFLD most attention has to be focused on the detection of advanced fibrosis (F3–F4) indicating the need for specific monitoring of complications such as portal hypertension and surveillance for hepatocellular carcinoma. The majority of our patients have been recruited from primary care physician practices. In this regard, our patient population is comparable to a recently published prospective study where advanced fibrosis was found in 9 of 134 liver biopsies (7%) performed in a middle-aged cohort of male and female US army personnel recruited from a primary care clinic [27]. An algorithm starting with the calculation of the FIB-4 index followed by calculating NIKEI only for patients with negative or indeterminate test results proved to be clinically very useful, since 82–84% of liver biopsies could have been avoided without missing a single case of advanced fibrosis in this low prevalence study population.

Several diagnostic panels have been developed to facilitate the noninvasive assessment of NAFLD and differentiation between different stages of disease. One approach utilizes the testing of biochemical markers of liver fibrosis: hyaluronic acid [8], [28], adiponectin [11], high-sensitivity CRP [9], type IV collagen [8], serum IL-6 [10], leptin [28], laminin [8], [28], c-peptide [7], and cytokeratin-18 [29], [19]. Using only a single biochemical marker to predict significant fibrosis has been evaluated in a few studies with inadequate diagnostic accuracy [14], [30]. In order to achieve a higher diagnostic accuracy in predicting fibrosis, scoring systems have been developed which combine multiple clinical and laboratory parameters, e.g. the FibroTest-FibroSURE® by Ratziu et al. [21], the OELF (**O**riginal **E**uropean **L**iver **F**ibrosis) score by Rosenberg et al. [15] and its modification, the ELF (**E**nhanced **L**iver **F**ibrosis) score by Ghua et al. [16]. However, the unavailability of the above mentioned serum markers in most centers prevent application of the proposed scoring systems on a day-to-day basis.

We developed and validated a simple noninvasive scoring system composed of routinely measured clinical and laboratory parameters to predict the presence or absence of advanced fibrosis in patients with NAFLD and BMI ranging from 18.8 to 71.8 kg/m^2^. This NIKEI consists of four variables (age, AST, AST/ALT ratio, and total bilirubin) and was highly accurate in predicting the absence of advanced fibrosis reaching negative predictive values of 99–100%.

The prevalence of NAFLD and NAFLD-related fibrosis increases with age. In addition to this association, older patients with NAFLD have a higher likelihood of disease progression or mortality [31], [32]. Our study results confirm this observation. An older age was highly significant associated with the presence of advanced fibrosis (p<0.001). It has previously been described, that NAFLD is more prevalent in cohorts of patients with pre-existing metabolic conditions than in the general population [1]. Our results confirm this observation as many patients of the study population were overweight/obese with further features of the metabolic syndrome. However, none of these conditions showed a statistically significant association with advanced fibrosis. Bilirubin levels were within the normal range. However we found bilirubin to be an independent predictor for advanced fibrosis in patients with NAFLD. These results are in line with findings from other studies [17]; [21]; [33]; [34]. Although bilirubin is not included in the NAFLD fibrosis score, it showed to be an independent predictor in univariate analysis [17]. Ratziu et al. and Stepanova et al. found bilirubin to be an independent predictor of significant fibrosis in patients with NAFLD in univariate and multivariate analysis [21], [34].

Among several algorithms, the NAFLD fibrosis score and the FIB-4 index have been validated most widely [19], [23]. Angulo et al. developed the “NAFLD fibrosis score”, a panel comprising six variables of age, glycaemia, BMI, platelet count, albumin and AST/ALT ratio. In the original study, the score had a NPV of 93% and 88% for advanced fibrosis in the estimation and validation groups, respectively. The AUROC was 0.84, and application of this model to the study population would have avoided liver biopsy in 75% of patients, with a correct prediction in 90% [17]. The evaluation in our study population showed an AUROC of 0.96, PPV of 100% and NPV of 96% (table 3). However the false negative rate was 25%, too high to be clinically useful.

The FIB-4 index, developed by Sterling et al. [26] from a database of 832 HIV/HCV coinfected patients, combines age, AST, ALT and platelet count and has been validated in NASH patients for the detection of fibrosis stages F3 and F4 with an AUROC of 0.802, and NPV and PPV of 90% and 80%, respectively [19].

Compared to the NAFLD fibrosis score and the FIB-4 index we achieved a better NPV with almost 100% and a better test performance with an AUROC of 0.96. Previously reported PPVs from the NAFLD fibrosis score and the validation of FIB-4 in NAFLD patients (86% and 80% respectively) were higher than in our study (60% in the estimation and 67% in the validation group), which may be due to differences in the prevalence of advanced fibrosis. However, if the clinical decision for closer follow-up and surveillance were based only on test results from these two scoring systems without liver biopsy, 8–23% of patients with advanced fibrosis would have been missed [17], [19], [23]. We achieved a lower misclassification rate by combining the FIB-4 and NIKEI indices in a stepwise fashion, which could possibly avoid inappropriate clinical management despite a large number of dispensable liver biopsies.

The AST/ALT ratio and the BARD score achieved worse results in our collective. Only 66% (AST/ALT ratio) and 56% (BARD score) would have been correctly classified, 27% (AST/ALT ratio) and 30% (BARD score) of the patients with advanced fibrosis would have been missed (see table S1 and figure S1).

However, the low prevalence of advanced fibrosis (8%) in our study population has to be taken into account when comparing our test results with other large series (prevalences of 26% [19] and 27% [17], respectively). This could have affected the high false negative rate of the tested scoring systems in our patient population.

In order to achieve a homogeneous study population truly consisting of patients only with NAFLD we had to exclude a large number of patients with preexisting diseases or accompanying medications, which may have introduced a selection bias. Due to the retrospective design of our study, clinical and laboratory data were incomplete in several cases, leaving only a limited number of patients to develop our scoring system as well as for the direct comparison with the well-established FIB-4 index. Furthermore, in order to increase the range of obesity and the number of patients with NASH in our study cohort we included cases from two tertiary liver centers in Germany with clearly different populations. While almost all patients recruited in Cologne were metabolic patients who had been referred to the outpatient department for further work-up after abnormal liver function tests had been detected by their treating primary care physicians, patients recruited in Essen, showed relatively normal liver enzymes, were severe obese and scheduled for bariatric surgery. Although the comparison of this two populations showed no statistically significant differences in laboratory or clinical parameters, especially concerning the prevalence of advanced fibrosis (see table 1), we can not exclude a potential impact on the score and its accuracy due to differences in pathogenic mechanisms among this two patient groups. This makes it necessary to confirm our results in further studies with a more homogeneous study population. Liver biopsy specimens were assessed by two experienced hepatopathologists who followed established histopathological criteria for the diagnosis and staging of NAFLD [24]; [25]. Histological slides were reexamined at a different time point by the same pathologists, who were blinded to all clinical and laboratory data. Inter and intra variability could have caused a bias.

Recently an algorithm for the work-up of patients with NAFLD has been proposed that incorporates FibroScan® for patients with indeterminate or high risk according to the NAFLD fibrosis score [35]. Although measuring liver stiffness with FibroScan® has been shown to produce reliable results in diagnosing advanced fibrosis in patients with various chronic liver diseases including NAFLD, this method reaches its limits in overweight and obese patients where as much as 20% of measurements are uninterpretable [36].

In summary, we developed and validated a simple noninvasive scoring system, the NAFLD-NIKEI, which relies on only four routinely measured laboratory parameters and as such has the potential to be used in every day practice. Our results suggest, that this index can reliably exclude advanced fibrosis in a large proportion of subjects with NAFLD. In combination with the FIB-4 index, misclassification with inadequate clinical management can be avoided while the need for liver biopsies can be reduced by more than 80%. Therefore, if our results can be confirmed in independent study samples from community dwelling patient populations with obesity and/or elevated liver enzymes and comparably low prevalence of advanced fibrosis, the proposed algorithm could play an important role in targeting the most appropriate candidate for liver biopsy.

## Supporting Information

Figure S1 **a.** ROC-curve analysis for the prediction of advanced fibrosis with *AST/ALT ratio >0.8 (n = 266)* in our study population. **b.** ROC-curve analysis for the prediction of advanced fibrosis with *BARD score (n = 242)* in our study population.
(TIF)

Table S1 **a.** Cut-off values and the classification of patients on the basis of the *AST/ALT ratio >0.8 (n = 266)*. A patient with a value below the lower cut-off level was classified as healthy (no advanced fibrosis), with a value above the upper cut-off level as morbid (advanced fibrosis). Patients with values between the cut-off levels are intermediate/not classifiable. **b.** Cut-off values and the classification of patients on the basis of the *BARD score (n = 242)*. A patient with a value below the lower cut-off level was classified as healthy (no advanced fibrosis), with a value above the upper cut-off level as morbid (advanced fibrosis). Patients with values between the cut-off levels are intermediate/not classifiable.
(DOC)
