# Supplementary figures and images for: NIKEI: A New Inexpensive and Non-Invasive Scoring System to Exclude Advanced Fibrosis in Patients with NAFLD
Source: PLoS One. 2013 Mar 26;8(3):e58360. doi: 10.1371/journal.pone.0058360 (PMC3608644; doi:10.1371/journal.pone.0058360)

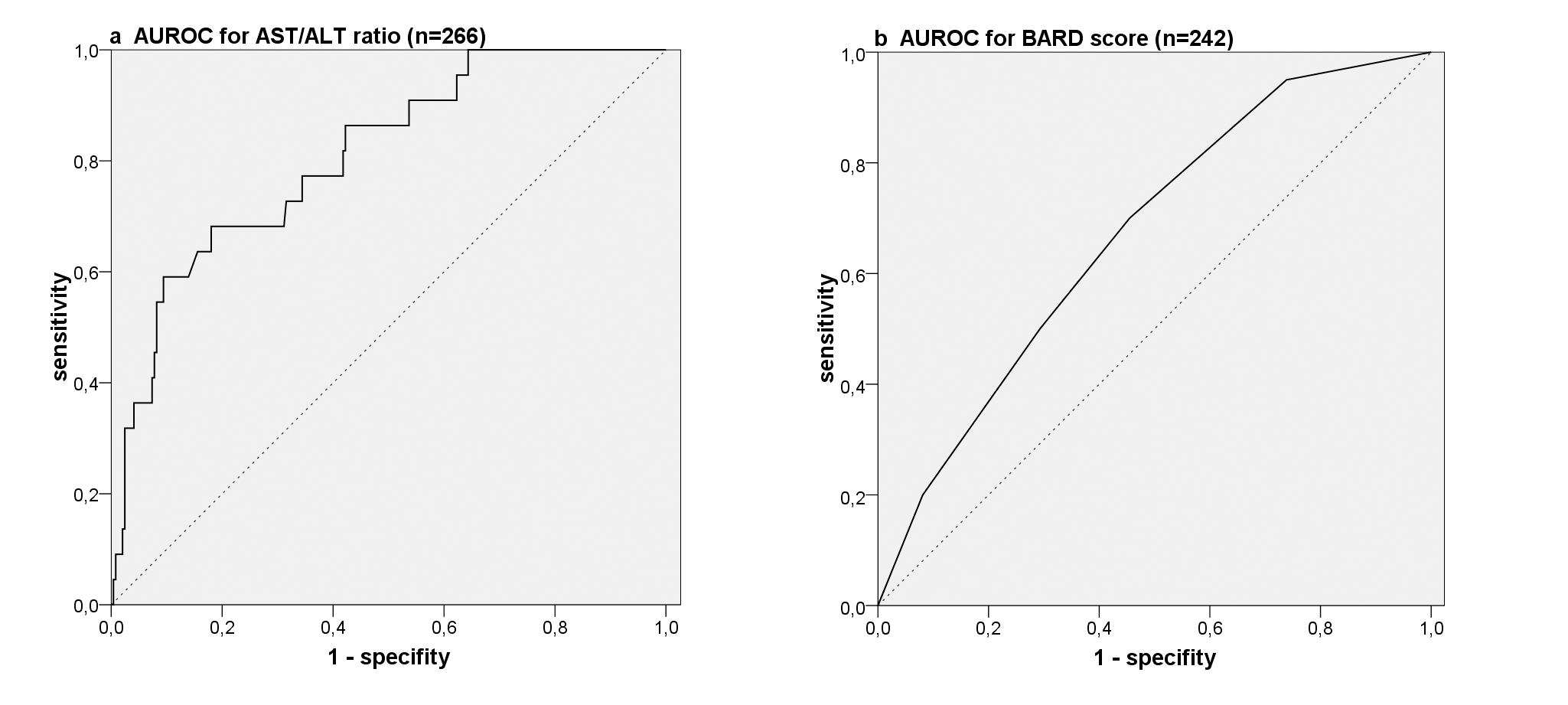

Supplement: Figure S1 — a. ROC-curve analysis for the prediction of advanced fibrosis with AST/ALT ratio >0.8 (n = 266) in our study population. b. ROC-curve analysis for the prediction of advanced fibrosis with BARD score (n = 242) in our study population. (TIF) [file pone.0058360.s001.tif]
