# Supplementary material for: NIKEI: A New Inexpensive and Non-Invasive Scoring System to Exclude Advanced Fibrosis in Patients with NAFLD
Source: PLoS One. 2013 Mar 26;8(3):e58360. doi: 10.1371/journal.pone.0058360 (PMC3608644; doi:10.1371/journal.pone.0058360)
Supplement: Table S1 — a. Cut-off values and the classification of patients on the basis of the AST/ALT ratio >0.8 (n = 266). A patient with a value below the lower cut-off level was classified as healthy (no advanced fibrosis), with a value above the upper cut-off level as morbid (advanced fibrosis). Patients with values between the cut-off levels are intermediate/not classifiable. b. Cut-off values and the classification of patients on the basis of the BARD score (n = 242). A patient with a value below the lower cut-off level was classified as healthy (no advanced fibrosis), with a value above the upper cut-off level as morbid (advanced fibrosis). Patients with values between the cut-off levels are intermediate/not classifiable. (DOC) [file pone.0058360.s002.doc]

**Supplemental tables S1:**

a) Diagnostic performance of the *AST/ALT ratio >0.8 (n=266)*

| AST/ALT ratio | ≤ 0.8 | 0.81-0.99 | ≥ 1 | All patients |
| --- | --- | --- | --- | --- |
| All patients, *n (%)* | 168 (63.2) | 46 (17.3) | 42 (19.5) | 266 |
| F0-F2, *n (%)* | 162 (66.4) | 44 (18.0) | 38( 15.6) | 244 |
| F3-F4, *n (%)* | 6 (27.3) | 2 (9.1) | 14 (63.6) | 22 |
| Sensitivity, *%* | 72.7 |  | 63.6 |  |
| Specifity, *%* | 66.4 |  | 84.4 |  |
| PPV, *%* | 16.3 |  | 26.9 |  |
| NPV, *%* | 96.4 |  | 96.3 |  |
| Likelihood-Ratio (+) | 2.1 |  | 4.1 |  |
| Likelihood-Ratio (-) | 0.41 |  | 0.43 |  |
| Diagnostic accuracy   0.66 | | | | |
| AUC (95% CI) 0.81 (0.72; 0.90) | | | | |

b) Diagnostic performance of the BARD score (n=242)

| BARD score | 0-1 |  | 2-4 | All patients |
| --- | --- | --- | --- | --- |
| All patients, n (%) | 127 (52.5) |  | 115 (47.5) | 242 |
| F0-F2, n (%) | 121 (54.5) |  | 101 (45.5) | 222 |
| F3-F4, n (%) | 6 (30) |  | 14 (70) | 20 |
| Sensitivity, % |  | 67 |  |  |
| Specifity, % |  | 54 |  |  |
| PPV, % |  | 10 |  |  |
| NPV, % |  | 95 |  |  |
| Likelihood-Ratio (+) |  | 1.5 |  |  |
| Likelihood-Ratio (-) |  | 0.61 |  |  |
| Diagnostic accuracy   0.56 | | | | |
| AUC (95% CI) 0.67 (0.55; 0.78) | | | | |

Supplemental tables S1: Cut-off values and the classification of patients on the basis of the a) *AST/ALT ratio >0.8 (n=266)* and b*) BARD (n=242)* score, respectively. A patient with a value below the lower cut-off level was classified as healthy (no advanced fibrosis), with a value above the upper cut-off level as morbid (advanced fibrosis). Patients with values between the cut-off levels are intermediate/ not classifiable.
